# Supplementary material for: Cell-autonomous Hedgehog signaling controls Th17 polarization and pathogenicity
Source: Nat Commun. 2022 Jul 14;13:4075. doi: 10.1038/s41467-022-31722-5 (PMC9281293; doi:10.1038/s41467-022-31722-5)
Supplement: Supplementary file 1 — Supplementary Information [file 41467_2022_31722_MOESM1_ESM.pdf]

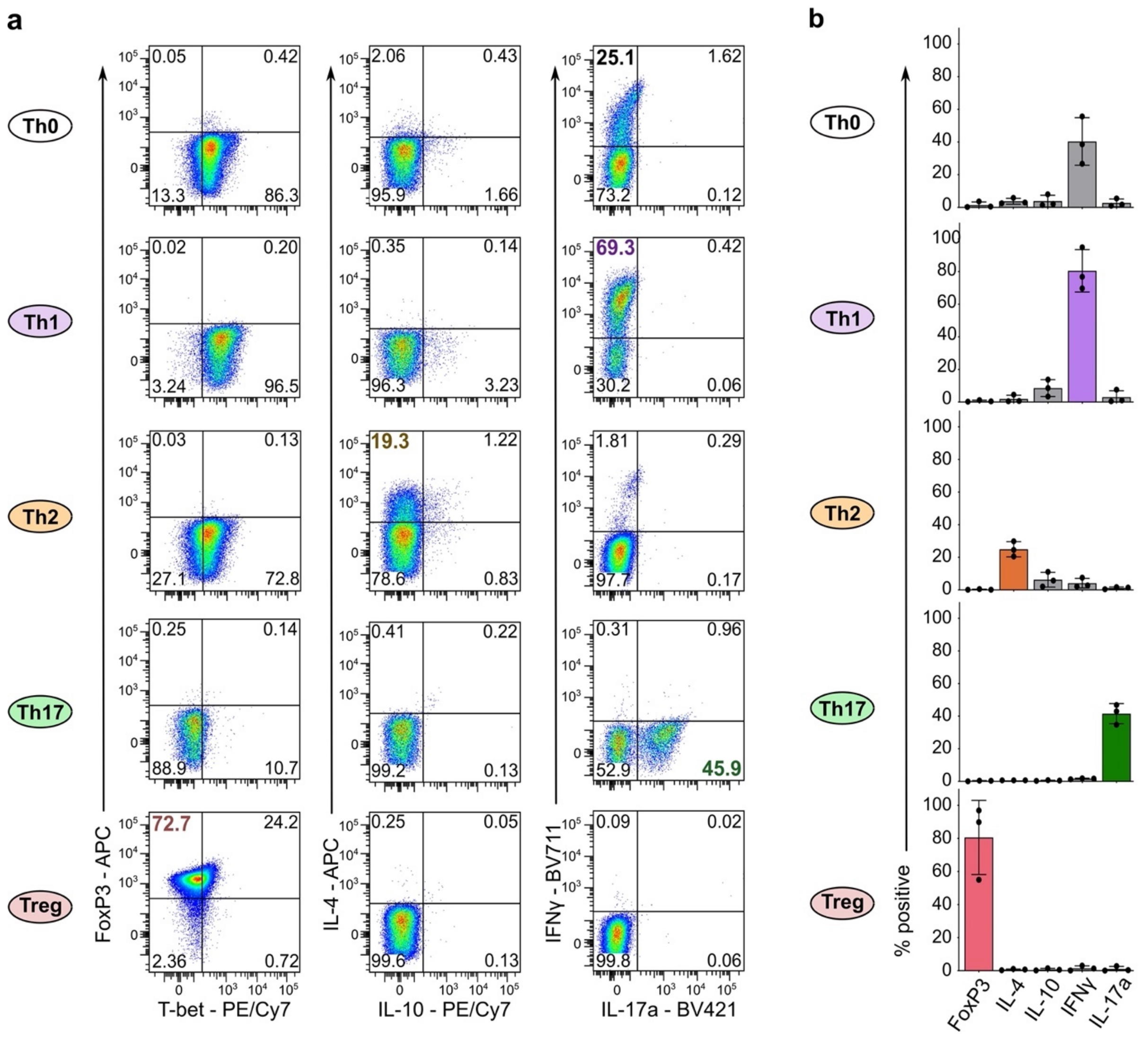

Supplemental Figure 1: T helper polarization *in vitro.*

Naïve CD4^{+} T cells were purified from spleen and peripheral lymph nodes of C57BL/6 mice and stimulated with plate-bound anti-CD3ε/CD28 antibodies in the presence of polarizing cytokines to generate Th0, Th1, Th2, Th17 and Treg subsets. Cells were assessed for FoxP3, T-bet, IL-4, IL-10, IL-17a and IFNγ production by flow cytometry on day 5. (**a**) Representative flow cytometry plots are shown. (**b**) Quantification of flow cytometry plots. n = 3 independent experiments. Source data are provided in the source data file.

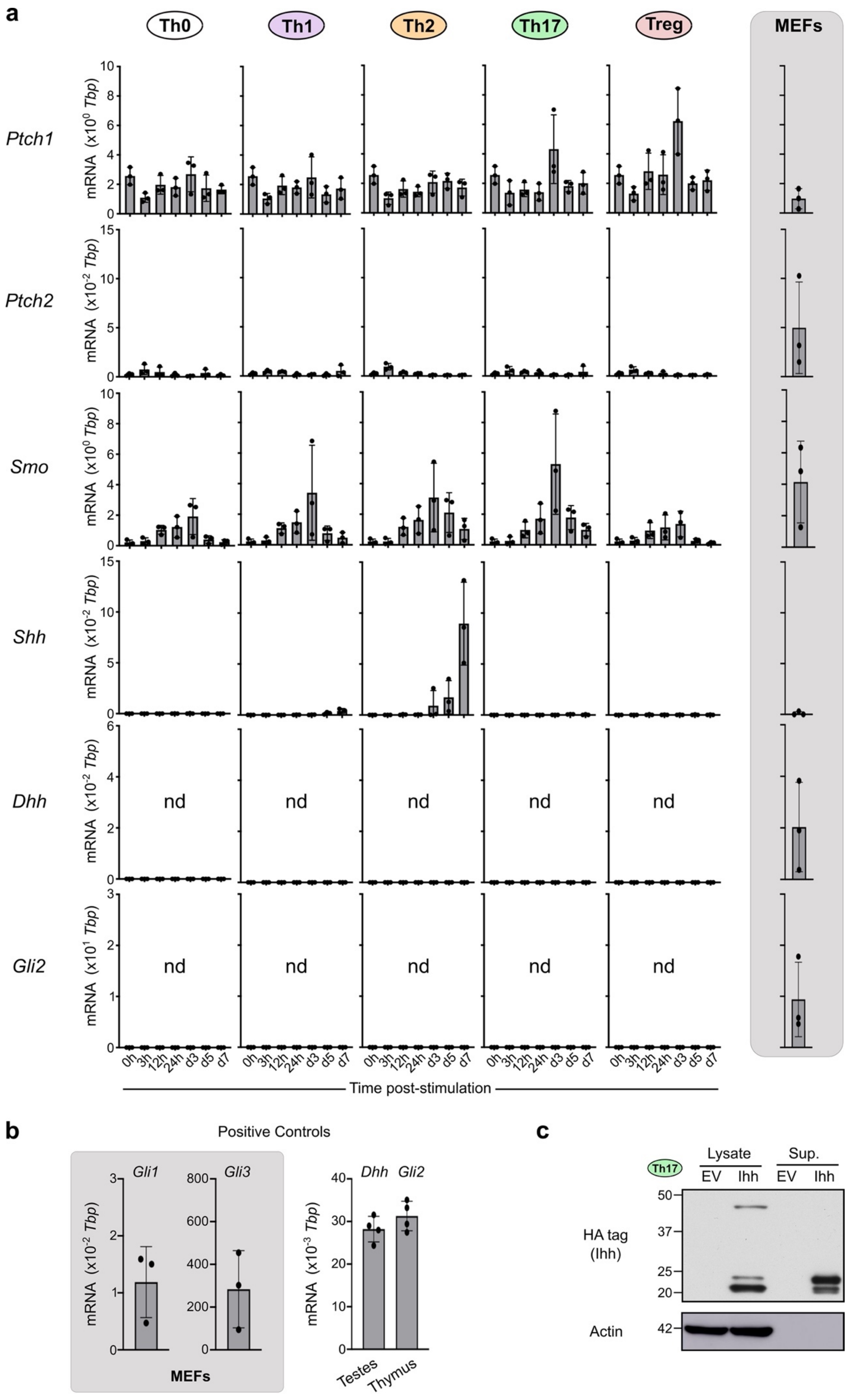
a
Th0
Th1
Th2
Th17
Treg
MEFs
Ptch1
Ptch2
Smo
Shh
Dhh
Gli2
mRNA (x10^0 Tbp)
mRNA (x10^-2 Tbp)
mRNA (x10^0 Tbp)
mRNA (x10^-2 Tbp)
mRNA (x10^-2 Tbp)
mRNA (x10^1 Tbp)
nd
0h 3h 12h 24h d3 d5 d7
Time post-stimulation
b
Positive Controls
Gli1
Gli3
mRNA (x10^-2 Tbp)
MEFs
Dhh
Gli2
mRNA (x10^-3 Tbp)
Testes
Thymus
c
Th17
Lysate
Sup.
EV
Ihh
EV
Ihh
HA tag
(Ihh)
Actin
50
37
25
20
42

Supplemental Figure 2: Expression of Hh signaling components during CD4$^+$ T helper cell polarization.

(**a**) Naïve CD4$^+$ T cells were purified from spleen and peripheral lymph nodes of C57BL/6 mice and stimulated with plate-bound anti-CD3ε/CD28 antibodies in the presence of polarizing cytokines to generate Th0, Th1, Th2, Th17 and Treg subsets. Expression of *Ptch1, Ptch2, Smo, Shh, Dhh* and *Gli2* were assessed by qRT-PCR in Th subsets at the indicated timepoints after TCR stimulation in the presence of polarizing cytokines. Data is normalized to *Tbp* as a reference gene. Similar results were obtained when *CD3ε* was used as a reference gene. n = 3 independent experiments. *Gli2/Dhh* mRNA was undetectable across all conditions tested. Data are means +/- SD. nd = not detected. As a positive control, expression levels of Hh components in Mouse Embryonic Fibroblasts (MEFs) were assessed (right column). n = 3. Data is normalized to *Tbp* as a reference gene. n = 3 independent experiments. (**b**) Left: Mouse Embryonic Fibroblasts (MEFs) were assessed for *Gli1* and *Gli3* expression by RT-qPCR. n = 3. Right: Testes and thymi were dissected from C57BL/6 mice (n=4), homogenized and lysed for qRT-PCR analysis of *Dhh* and *Gli2 mRNA expression,* respectively. (**c**) Th17 cells were transduced with pMIG retrovirus encoding HA-(N-terminal)-tagged Ihh or empty vector (EV) control on day 1 post stimulation. Cells (Lysate) and culture supernatants (Sup.) were harvested separately on day 3 for immunoblot analysis of HA and Actin. Representative blot of 2 independent experiments with 2 separate mice each is shown. (**a/c**) MEF data is shaded in gray. Source data are provided in the source data file.

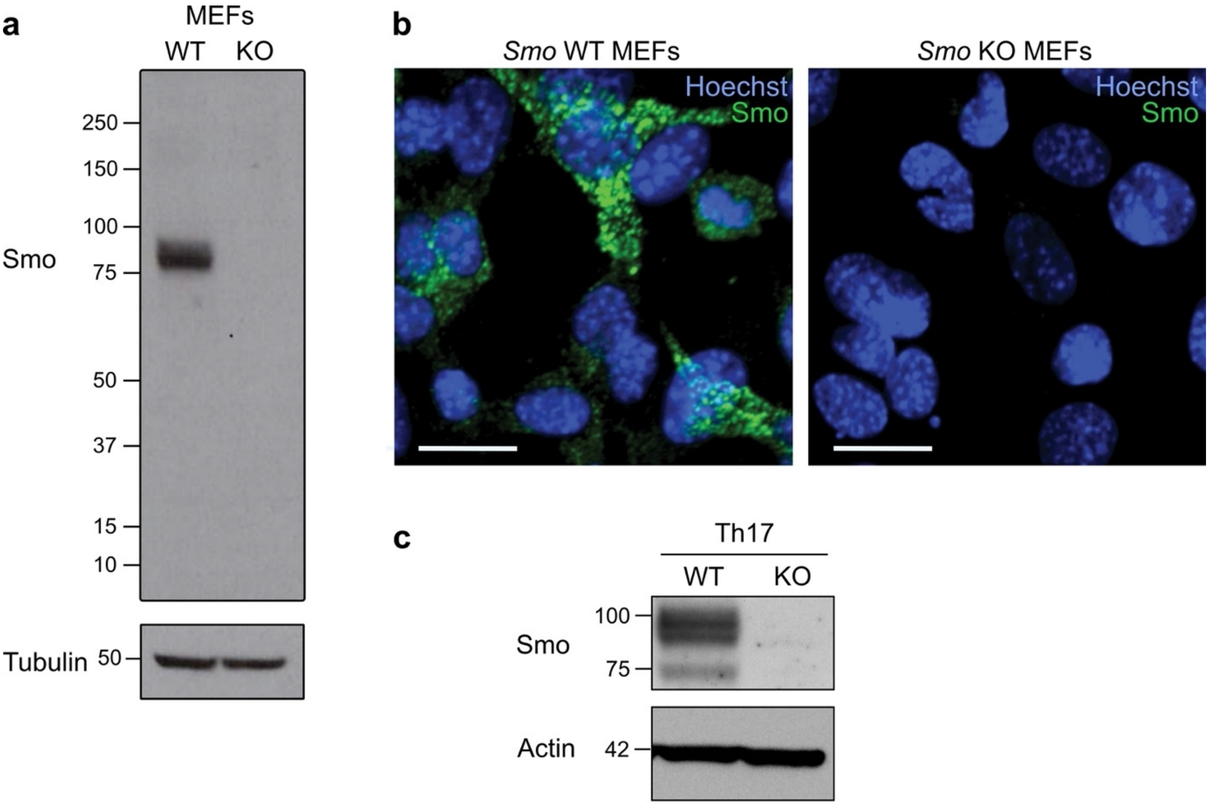

Supplemental Figure 3: Validation of novel monoclonal antibodies raised against the C-terminus of murine Smo (mcSmo).
(**a, b**) Wildtype (WT) or *Smo* knockout (KO) Mouse Embryonic Fibroblasts (MEFs) were used for validation of novel monoclonal Smo antibodies raised against murine Smo. (**a**) Immunoblot analysis of Smo in WT and KO MEFs using mcSmo. n = 2 independent experiments. (**b**) Immunofluorescence imaging of WT and KO MEFs labelled with mcSmo (green). Nuclei were stained with Hoechst (blue). n = 3 independent experiments. Scale bars: 20µm. (**c**) Immunoblot analysis of Smo expression in Th17 cells polarized from spleens of tamoxifen-treated $CD4ER^{T2}Cre$ $Smo^{+/+}$ (WT) and $CD4ER^{T2}Cre$ $Smo^{fl/fl}$ (KO) mice. n = 2 independent experiments with 2-3 mice per condition. Source data are provided in the source data file.

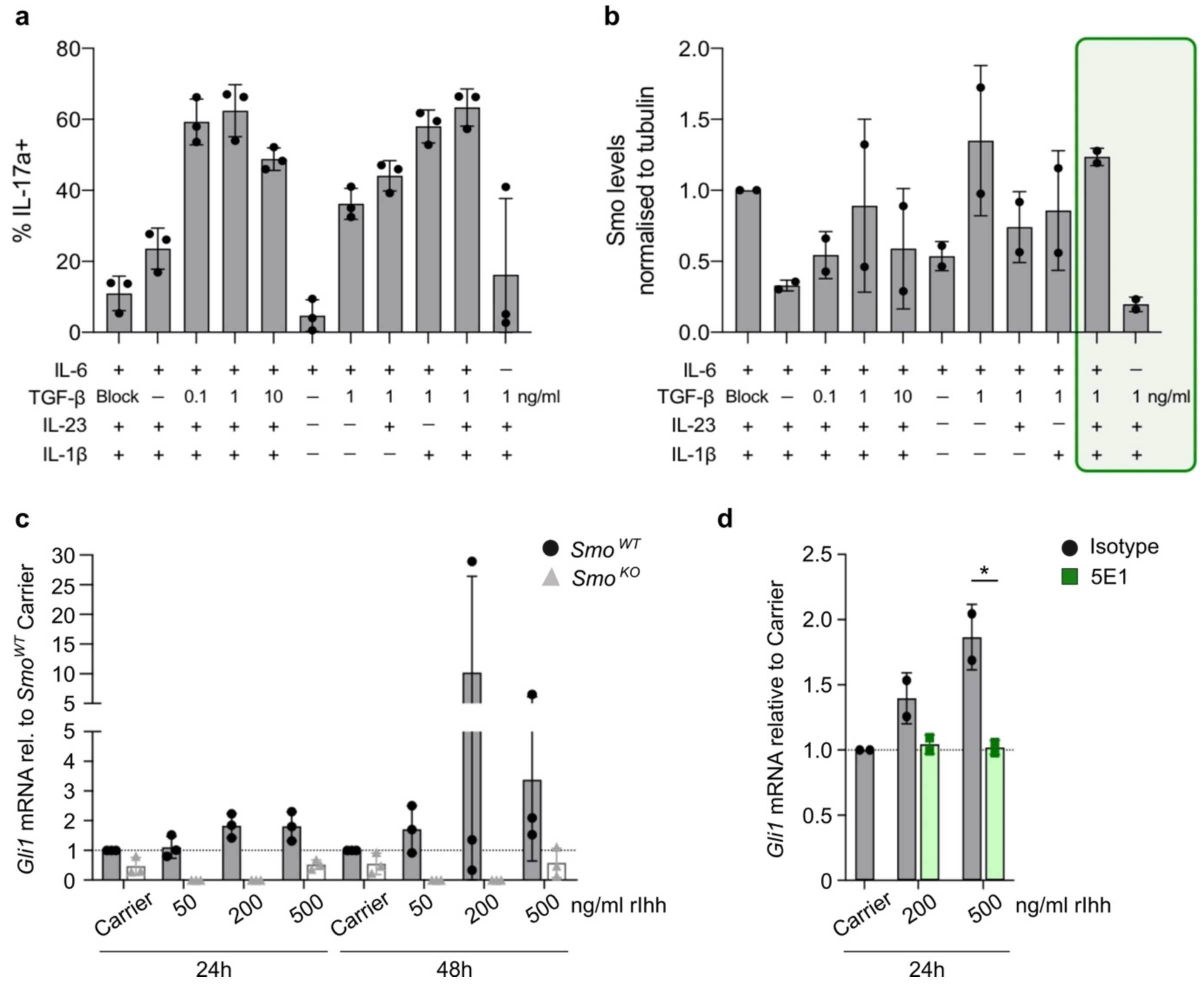

Supplemental Figure 4: Expression of IL-17a expression in different Th17-polarizing conditions, densitometric quantification of Smo Western blots and validation of recombinant Ihh and 5E1 blocking antibody.

(**a, b**) Naïve CD4$^+$T cells were purified from spleen and peripheral lymph nodes of C57BL/6 mice and stimulated with plate-bound anti-CD3ε/CD28 antibodies in the presence of the indicated polarizing cytokines or anti-TGFβ blocking antibody (Block). (**a**) Cells were assessed for IL-17a production by flow cytometry on day 5. Quantification of flow cytometry plots is shown. n = 3 independent experiments (**b**) Cells were assessed for Smo and α-tubulin protein levels by Western Blot at day 3. Data shows densitometric expression of Smo protein levels normalized to α-tubulin expression relative to the TGF-β blocking antibody-treated condition. The full Th17-polarising conditions +/- IL-6 are highlighted in green shading. n = 2. (**c, d**) MEFs were cultured to confluency. When confluent, cells were treated with the indicated concentrations of active recombinant Indian Hedgehog N-terminal peptide. 24h and 48 hours after stimulation cells were lysed and RNA was extracted for mRNA levels of *Gli1* and *Tbp*. *Gli1* mRNA is used as a reporter of active Hh signalling. (**c**) *Smo* wildtype (WT) and *Smo*$^{-/-}$ (KO) mouse embryonic fibroblasts (MEFs) were treated as described above. *Smo* KO MEFs, which are unable to induce canonical Hh signalling, serve as a specificity control. n = 3 independent experiments. (**d**) Wildtype MEFs were treated as described above for 24h either in the presence of 10µg/ml Hh ligand blocking antibody 5E1 or isotype control. n = 2 independent experiments. Data are means +/- SD. p-values were calculated using an unpaired two-tailed Student's t test. * p<0.05. Source data are provided in the source data file.

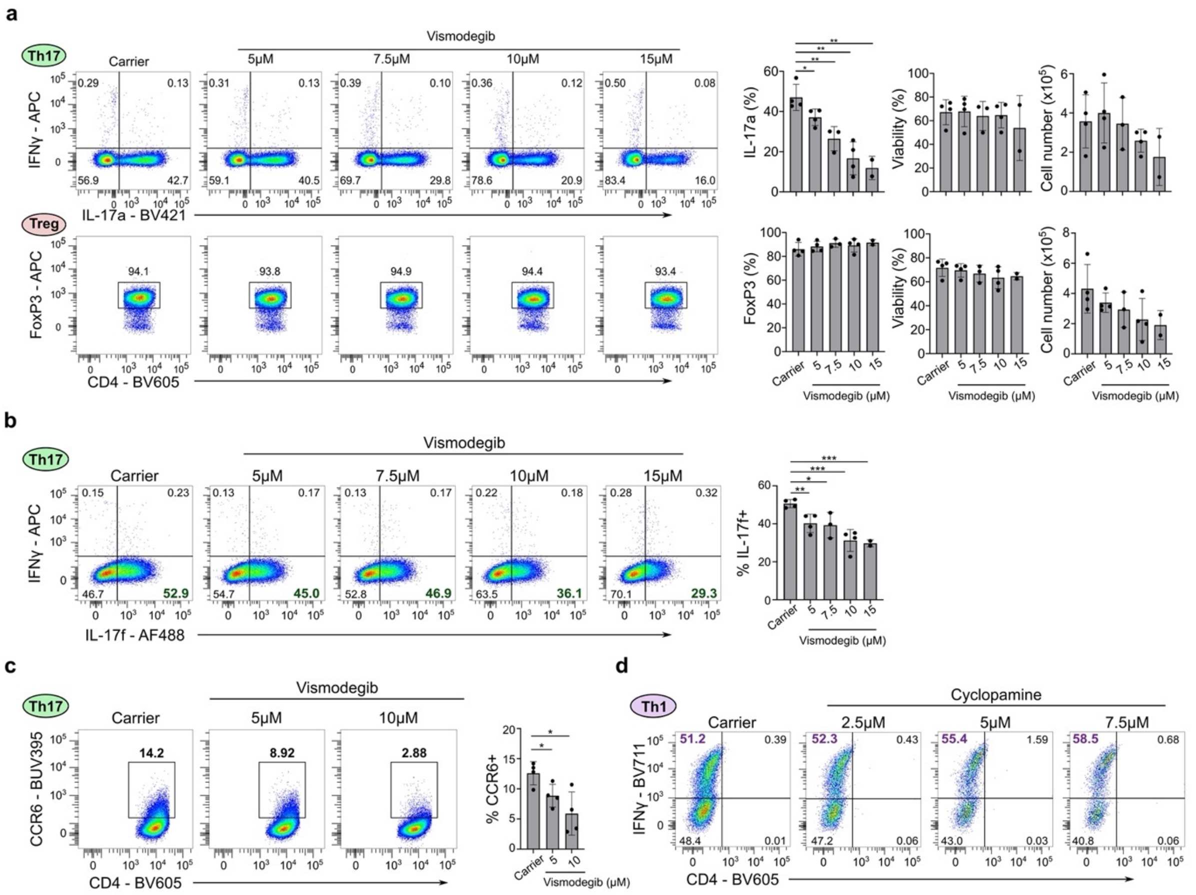

Supplemental Figure 5: Clinically-approved small molecule Hedgehog inhibitor vismodegib selectively blocks Th17 polarization *in vitro*.
(**a**-**c**). Naïve $CD4^+$ T cells were stimulated under Th17 (top row) or Treg (bottom row) polarizing conditions in the presence of the indicated doses of vismodegib or carrier control for five days. Cells were harvested for analysis by flow cytometry on day 5. (**a**) Panel on the left shows representative flow cytometry plots. Quantitation of IL-17a/FoxP3 expression in Th17 cells and Tregs respectively, viability measured by absence of live/dead staining, and cell numbers are shown on the right. n = 2-4 independent experiments. (**b**) Panel on the left shows representative flow cytometry plots of IL-17f expression in Th17 cells. Quantitation shown on the right. n = 2-4 independent experiments. (**c**) Panel on the left shows representative flow cytometry plots of CCR6 expression in Th17 cells. Quantitation shown on the right. n = 4 independent experiments. (**d**) Naïve $CD4^+$ T cells were stimulated under Th1 polarizing conditions in the presence of the indicated doses of cyclopamine or carrier control as previously described. Cells were harvested for analysis by flow cytometry on day 5. Full dose titration was performed as n = 2. Data are means +/- SD. p-values were calculated using an unpaired two-tailed Student's t test. * p<0.05, ** p<0.01, *** p<0.001. Source data are provided in the source data file.

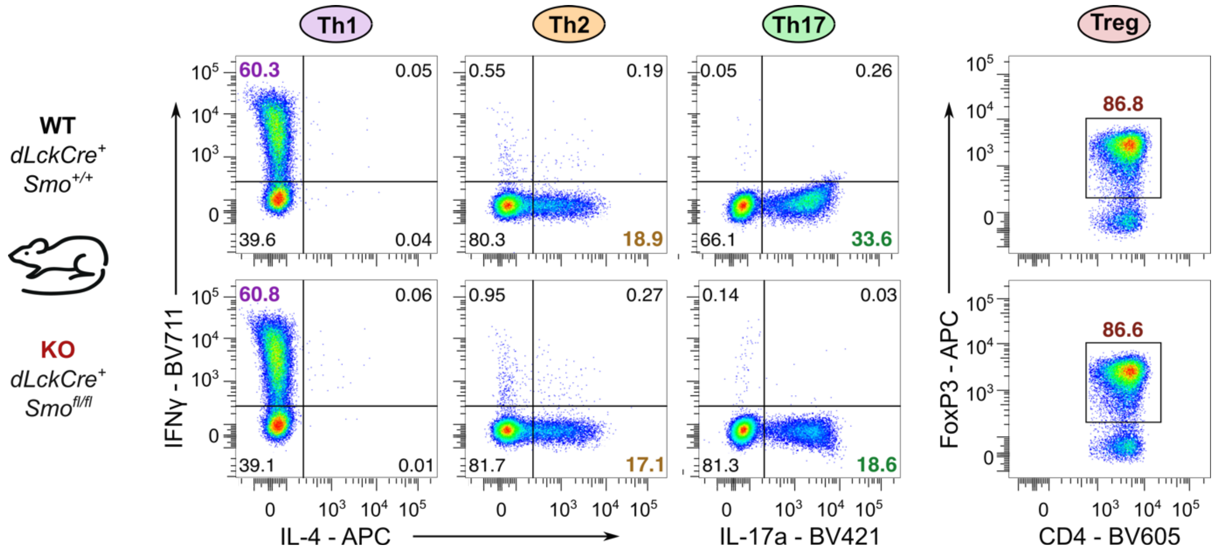

Supplemental Figure 6: Conditional knockout of *Smo* in CD4$^+$ T cells leads to diminished Th17 polarization but does not affect other Th lineages.
Naïve CD4$^+$ T cells were purified from spleen and peripheral lymph nodes of either *dLckCre*$^+$ *Smo*$^{+/+}$ (WT) or *dLckCre*$^+$ *Smo*$^{fl/fl}$ (KO) C57BL/6 mice. Cells were stimulated under Th1, Th2, Th17 or Treg polarizing conditions and harvested for analysis by flow cytometry on day 5. n=2-3 mice per genotype.

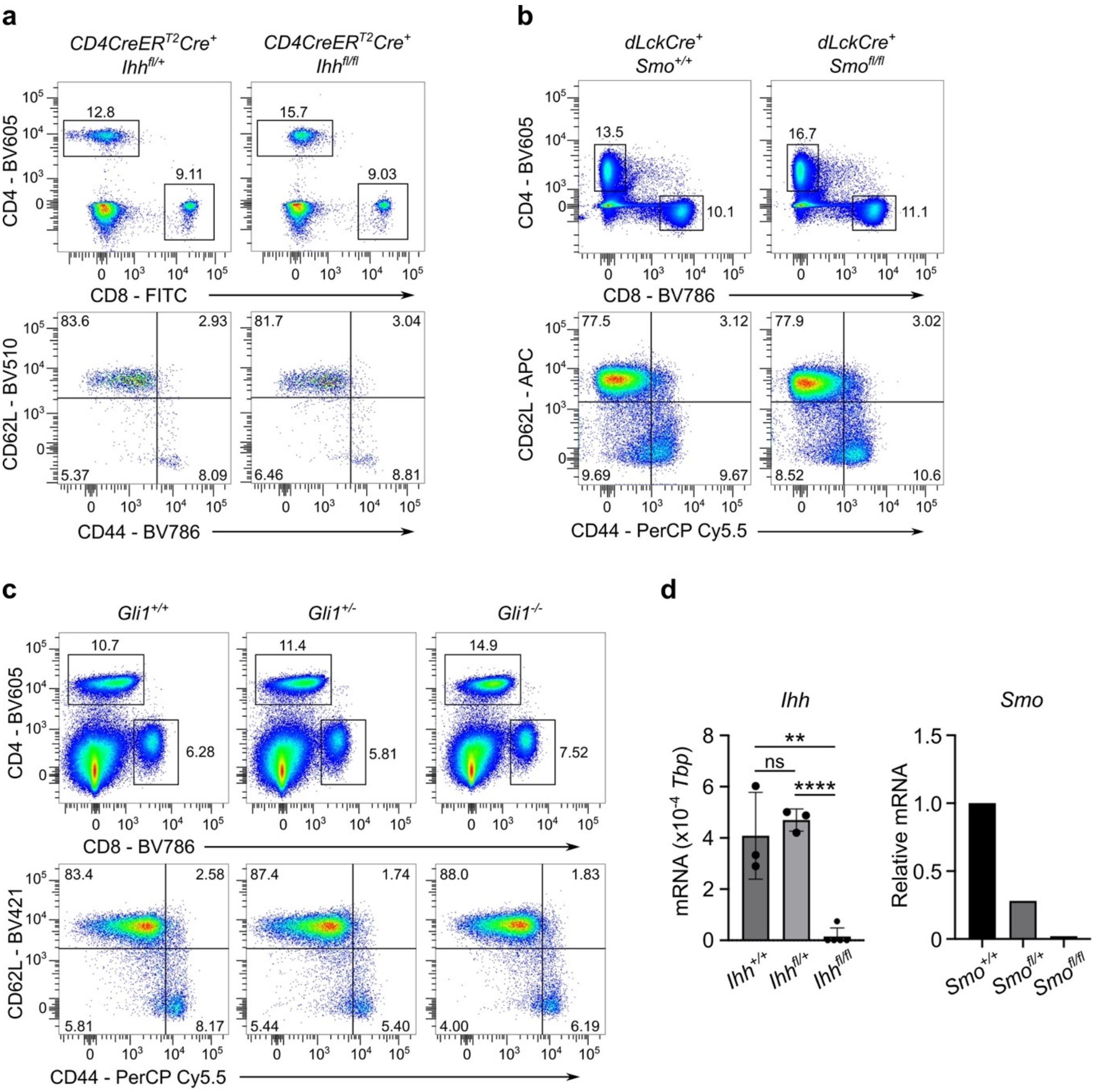

Supplemental Figure 7: Characterization and validation of conditional *Ihh* and *Smo* KO mice and constitutive *Gli1* KO mice.

(**a-c**) Spleens were mashed and strained through a 70µm filter prior to flow cytometric analysis of CD4, CD8, CD44 and CD62L expression by flow cytometry. Upper panels show CD4 and CD8 percentages of all cells. Lower panels show CD44 and CD62L expression of $CD4^+$ cells. (**a**) Phenotype of T cells from *$CD4^{ERT2}Cre^+$ $Ihh^{fl/+}$* and *$CD4^{ERT2}Cre^+$ $Ihh^{fl/fl}$* mice. (**b**) Phenotype of T cells from *$dLckCre^+$ $Smo^{+/+}$* and *$dLckCre^+$ $Smo^{fl/fl}$* mice. (**c**) Phenotype of T cells from *$Gli1^{+/+}$, $Gli1^{eGFP/+}$* and *$Gli1^{eGFP/eGFP}$* mice. Flow cytometry plots are representative of 3-6 mice analyzed per genotype. (**d**) RT-qPCR analysis of *Ihh* and *Smo* in T cells from *$dLckCre^+$ Ihh^{fl}* and *$dLckCre^+$ Smo^{fl}* mice, respectively. n=1-3 independent experiments. Data is normalized to *Tbp* as a reference gene. Similar results were obtained when *$CD3\varepsilon$* was used as a reference gene. p-values were calculated using an unpaired two-tailed Student's t test. ** $p<0.01$, **** $p<0.0001$. Source data are provided in the source data file.

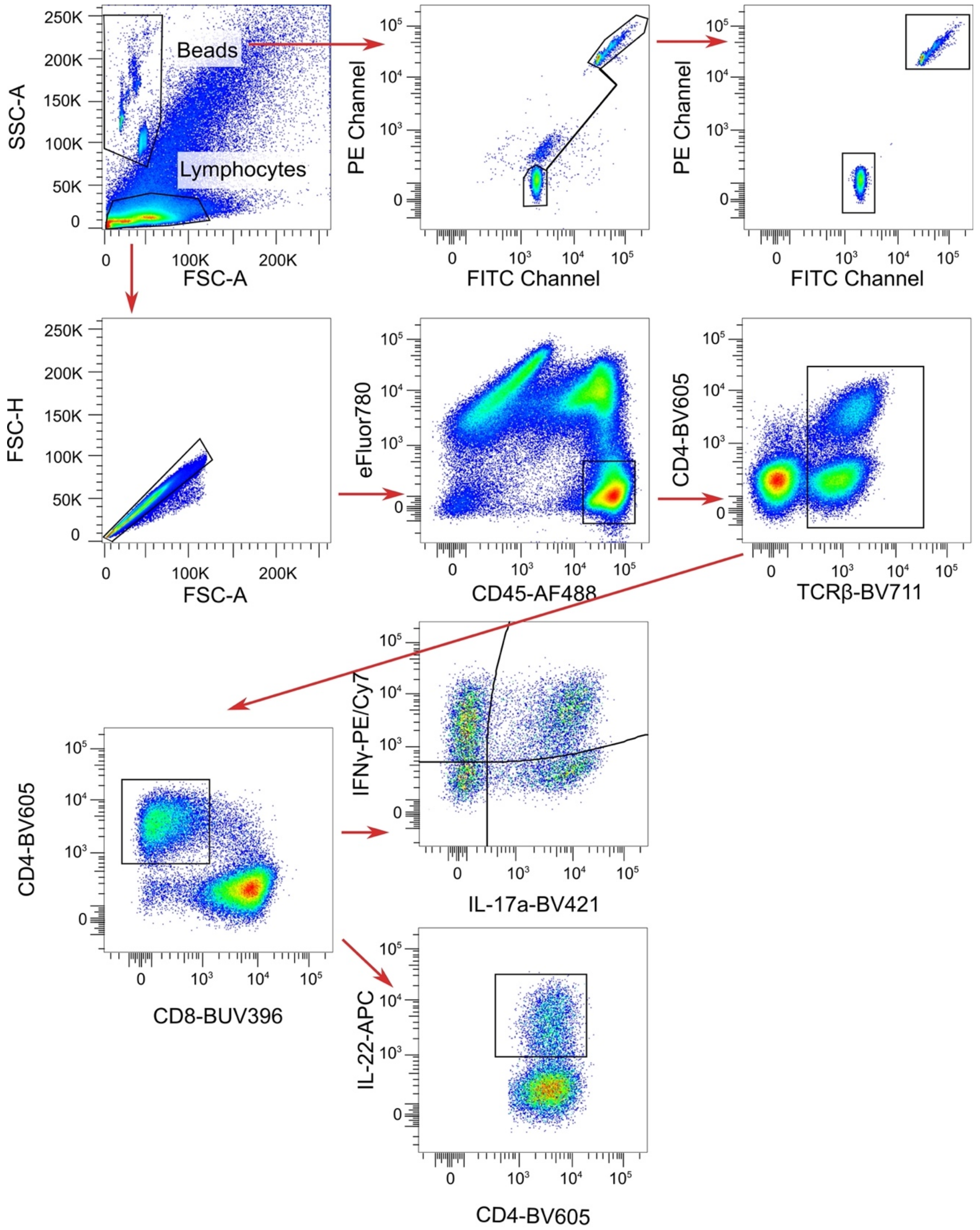

Supplemental Figure 8: Gating strategy for IELs from the small intestine.
Gating strategy adopted for identification and analysis of T cells and counting beads from small intestine preps harvested from mice treated with the anti-CD3 mAb injection model of small intestinal inflammation. Top row shows gating strategy used to identify the two populations of AccuCheck counting beads. Remaining plots show sequential gating from lymphocytes on FSC/SSC, to single cells, to live (eFluor780^{neg}) CD45^{+} cells, TCRβ^{+} cells and CD4^{+} CD8^{-} cells, respectively. Remaining plots show gating used to identify IFNγ^{+}, IL-17a^{+} and IL-22^{+} CD4^{+} T cells.

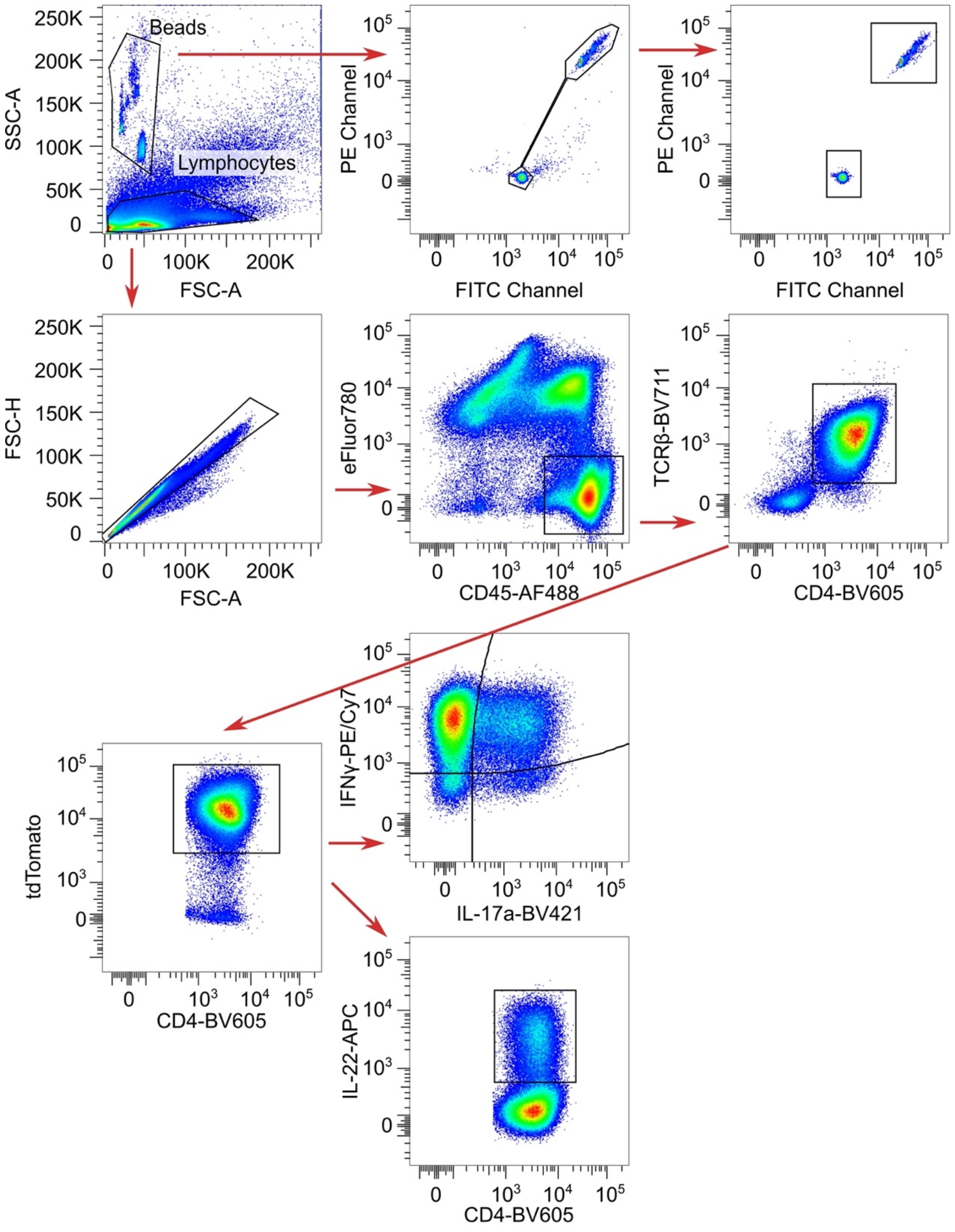

Supplemental Figure 9: Gating strategy for CD4⁺tdTom⁺ cells in the adoptive transfer colitis model.

Gating strategy adopted for identification and analysis of T cells and counting beads from colon preps harvested from recipient mice subjected to T cell adoptive transfer colitis. Top row shows gating strategy used to identify the two populations of AccuCheck counting beads. Remaining plots show sequential gating from lymphocytes on FSC/SSC, to single cells, to live (eFluor780 ^{neg}) CD45$^+$ cells, to CD4$^+$ TCRβ$^+$ cells and to tdTom$^+$ cells, respectively. Remaining plots show gating used to identify IFNγ$^+$, IL-17a$^+$ and IL-22$^+$ CD4$^+$ T cells.

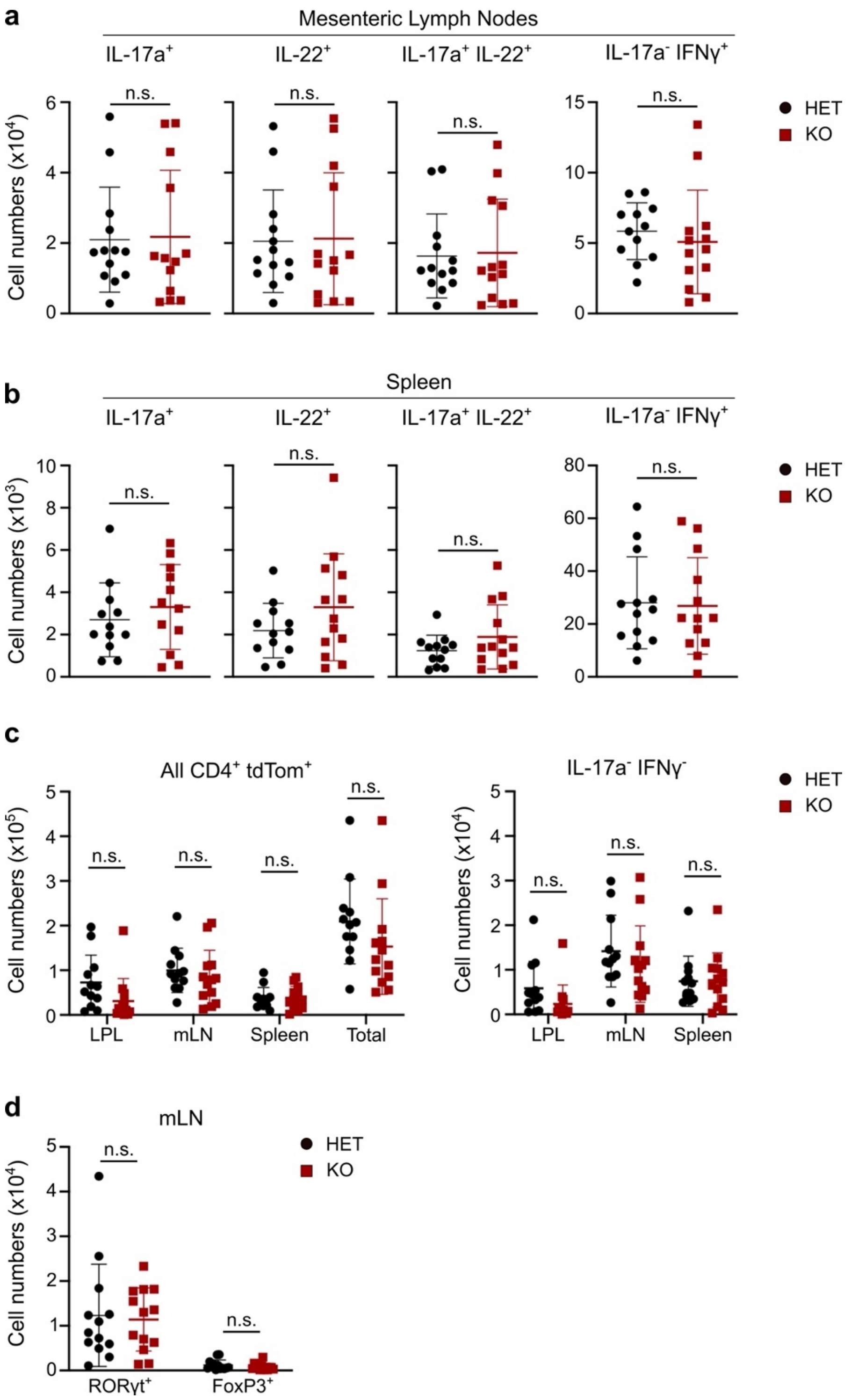

Supplemental Figure 10: Homeostasis and maintenance of CD4^{+} T cells is unaffected by loss of *Ihh* in the adoptive colitis model.

(**a-d**) *Rag2$^{-/-}$* mice were injected *i.p.* with 4x10^{5} CD45RB^{hi} CD25^{-} tdTom^{+} CD4^{+} T cells isolated from the spleens and peripheral lymph nodes of tamoxifen-treated *CD4ER^{T2}Cre Ihh$^{fl/+}$* (HET) and *CD4ER^{T2}Cre Ihh$^{fl/fl}$* (KO) mice. Numbers of IL-17a^{+}, IL-22^{+}, IL-17a^{+}/IL-22^{+}, IFNγ^{+}/IL-17a^{-}, IFNγ^{-}/IL-17a^{-}, RORγt^{+}, FoxP3^{+} CD4^{+} T cells isolated from lamina propria (LPL), mesenteric lymph node (mLN) and spleen are shown. (**a-c**) Gating strategy as shown in Suppl. Fig. 9. All cells are gated on tdTom^{+} CD4^{+} T cells. Data are means +/- SD. p-values were calculated using an unpaired two-tailed Student's t test. n.s. = not significant. Source data are provided in the source data file.

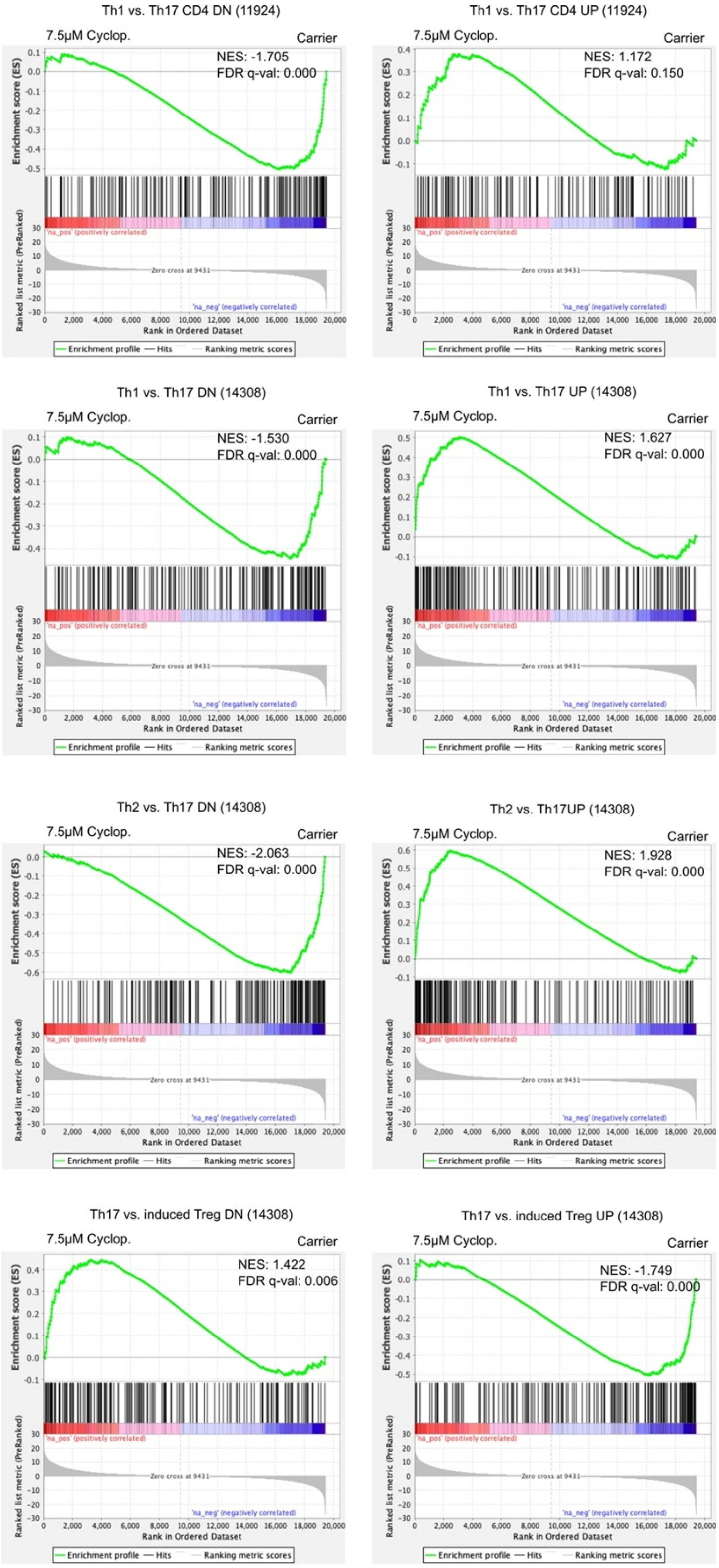

Supplemental Figure 11: Gene Set Enrichment Analysis of RNASeq data demonstrates loss of Th17 identity upon Hh inhibitor treatment.
Gene set enrichment plots (GSE11924 and GSE14308) using RNASeq data shown in Fig. 7d. Th17 cells were polarized as described, stimulated in the presence of the indicated dose of cyclopamine or carrier control for three days, and harvested on day 3. Six samples/group.

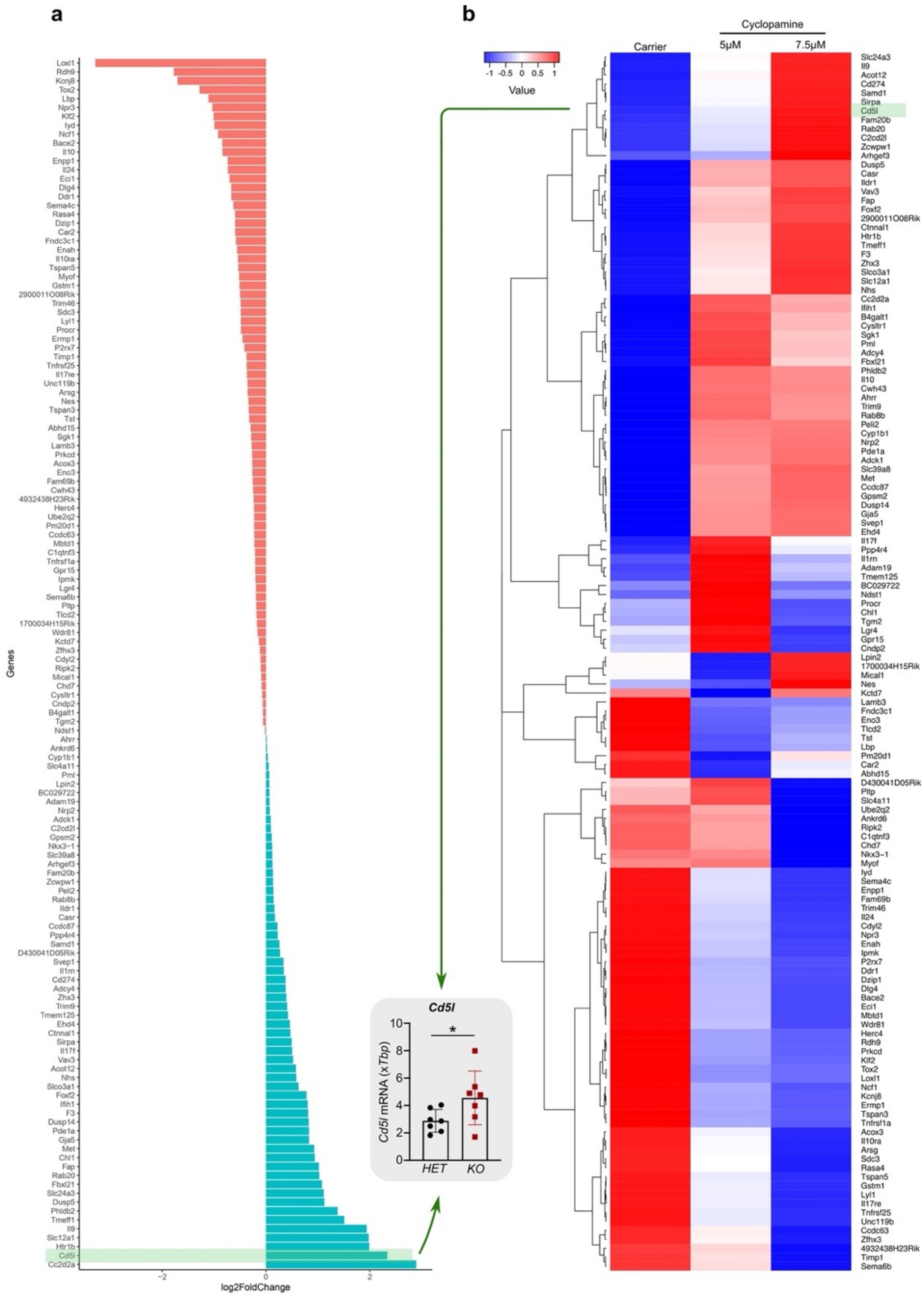

Supplemental Figure 12: Hh inhibitor treatment affects predicted, putative Gli3 target genes in Th17 cells.

RNASeq analysis of all putative Gli3 target genes as predicted by *Miraldi et al. (2019)*. Th17 cells were polarized as described above, stimulated in the presence of the indicated doses of cyclopamine or carrier control for three days and harvested at day 3. Six samples/group. (**a**) Analysis of putative Gli3 target gene expression between carrier and 5μM cyclopamine. *Cd5l* shaded in green. (**b**) Analysis of putative Gli3 target gene expression between carrier, 5μM and 7.5μM cyclopamine. *Cd5l* shaded in green. Grey-shaded inlay shows *Cd5l* mRNA expression as assessed by qRT-PCR from *in vitro* polarized Th17 cells harvested at day 3 isolated from tamoxifen-treated *CD4ER^{T2}Cre Ihh$^{fl/+}$* (HET) and *CD4ER^{T2}Cre Ihh$^{fl/fl}$* (KO) mice. Data is normalized to *Tbp* as a reference gene. Similar results were obtained when *CD3ε* was used as a reference gene. p-values were calculated using an unpaired one-tailed Student's t test. * = $p < 0.05$.n = 7 mice. Source data are provided in the source data file.

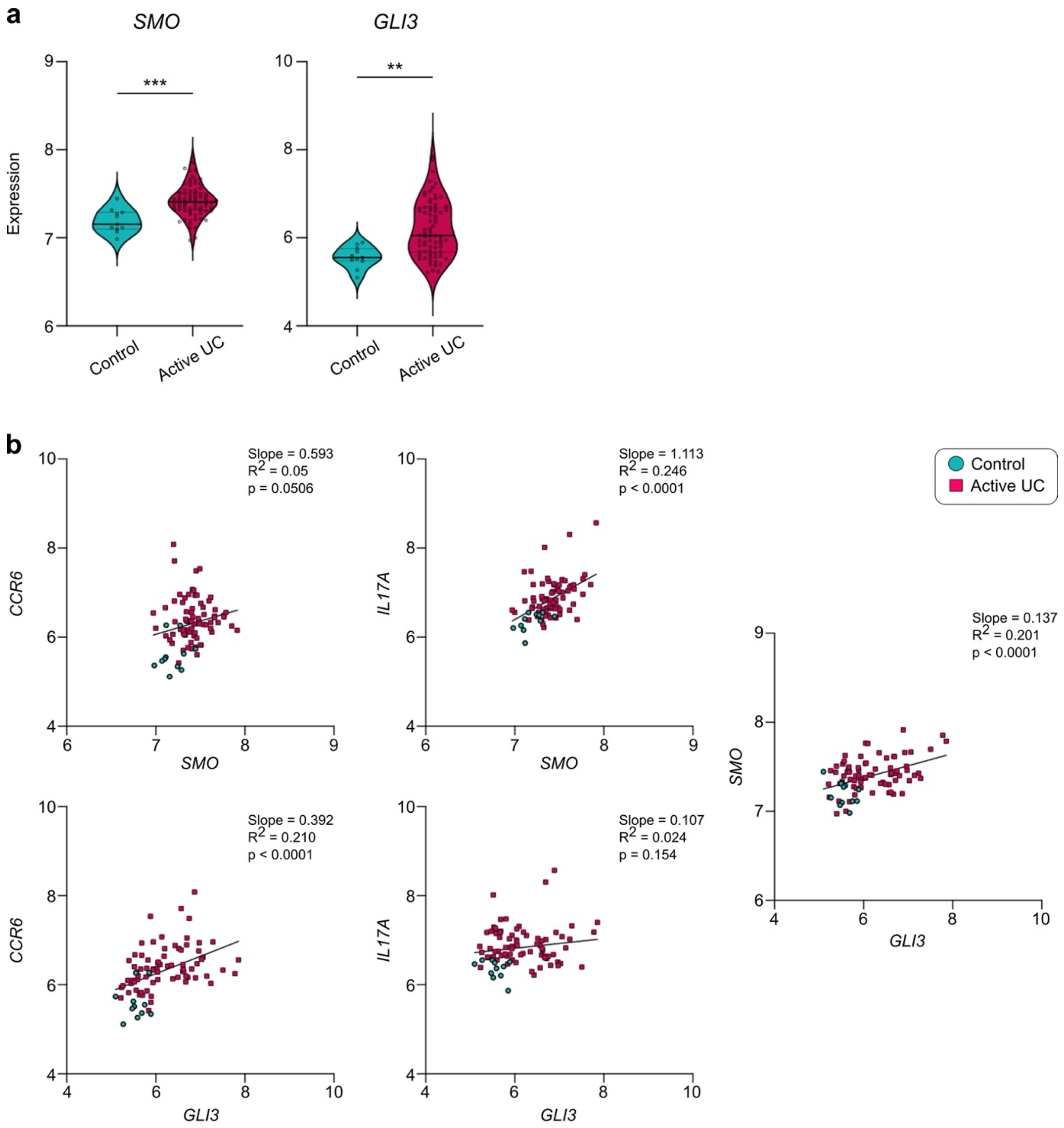

Supplemental Figure 13: Hedgehog signaling components are upregulated in an ulcerative colitis cohort and expression of *SMO* and *GLI3* correlate with markers of Th17 cell infiltration. (**a, b**) Expression of *SMO* and *GLI3* mRNA in human rectal biopsy samples from patients with active ulcerative colitis (UC) or healthy controls recruited as part of the RISK study (GSE117993). Each datapoint represents an individual patient. (**a**) shows expression of *SMO* and *GLI3* across all groups. p-values were calculated using a limma based moderated t test. ** p<0.01, *** p<0.001. (**b**) shows correlation analysis between *SMO* and *GLI3* and Th17-related transcripts *CCR6* and *IL17A* using simple linear regression analysis. Source data are provided in the source data file.

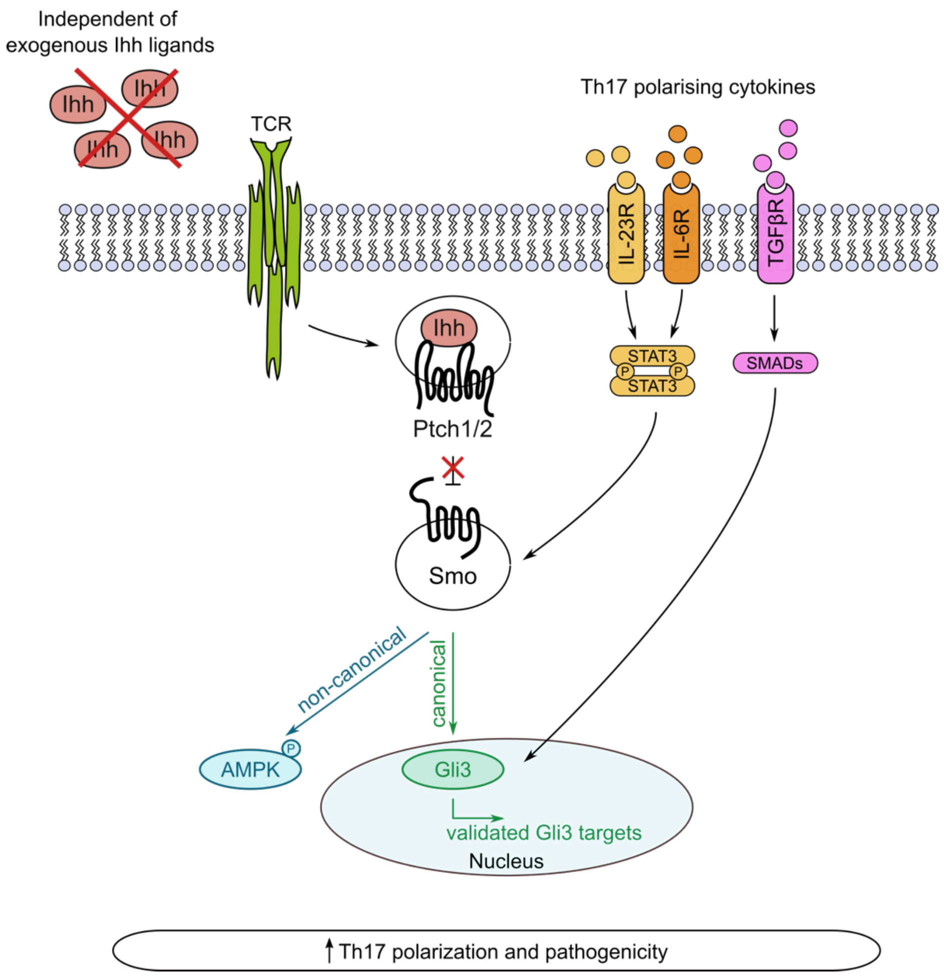

Supplemental Figure 14: Proposed model of Hh signaling in Th17 cells.
We show that upon TCR stimulation, Th17 polarizing cytokines upregulate the Hh components Smo and Gli3, allowing cell-endogenous Indian Hh (Ihh) ligand to activate the pathway through signal transducer Smo. Smo promotes Th17 polarization through both canonical activation of Hh transcription factor Gli3 and non-canonical AMPK activation *in vitro* and *in vivo*.

Lineage	Cytokine/Blocking Antibody	Final concentration	Cat no.	Supplier
Th0	IL-2	20ng/ml	200-02-500	Peprotech
Th1	IL-12	4ng/ml	210-12-10	Peprotech
Th1	Anti-IL-4	5µg/ml	504115	Biolegend
Th2	IL-4	10ng/ml	214-14-50	Peprotech
Th2	Anti-IFNɣ	10µg/ml	517904	Biolegend
Th17	IL-1β	10ng/ml	211-11B-50	Peprotech
Th17	IL-6	20ng/ml	216-16-50	Peprotech
Th17	IL-23	10ng/ml	1887-ML	R&D Systems
Th17	TGFβ	1ng/ml	100-21-50	Peprotech
Th17	Anti-IL-4	5µg/ml	504115	Biolegend
Th17	Anti-IFNɣ	10µg/ml	517904	Biolegend
iTreg	IL-2	20ng/ml	200-02-500	Peprotech
iTreg	TGFβ	10ng/ml	100-21-50	Peprotech
iTreg	Anti-IL-4	5µg/ml	504115	Biolegend
iTreg	Anti-IFNɣ	10µg/ml	517904	Biolegend

Table 1: Murine polarizing cytokines used for $CD4^+$ Th cell polarization

Target	Clone	Reactivity	Fluorochrome	Dilution	Cat no.	Supplier
CCR6	140706	Mouse	BUV395	1:100	747831	BD
CD3ε	145-2C11	Mouse	BUV395	1:50	563565	BD
CD4	RM4-5	Mouse	BV605	1:200	100548	Biolegend
CD4	RM4-5	Mouse	APC	1:200	100516	Biolegend
CD8a	53-6.7	Mouse	BV605	1:200	100744	Biolegend
CD8a	53-6.7	Mouse	PE	1:200	100707	Biolegend
CD8a	53-6.7	Mouse	BUV396	1:200	563786	BD
CD44	IM7	Mouse/Human	BV785	1:400	103059	Biolegend
CD45	30-F11	Mouse	Alexa Fluor 488	1:200	103122	Biolegend
CD45	30-F11	Mouse	PE/Cy7	1:200	103114	Biolegend
CD62L	MEL-14	Mouse	BV421	1:200	104436	Biolegend
CD90.1	HIS51	Mouse/Rat	APC	1:200	17-0900-82	BD
CD90.2	53-2.1	Mouse	PE	1:200	140308	Biolegend
TCRβ	H57-597	Mouse	BV711	1:200	109243	Biolegend
TCRγδ	GL3	Mouse	Alexa Fluor 488	1:200	118128	Biolegend
IL-4	11B11	Mouse	APC	1:100	504106	Biolegend
IL-4	11B11	Mouse	BV711	1:100	504133	Biolegend
IL-10	JES5-16E3	Mouse	PE/Cy7	1:100	505025	Biolegend
IL-17a	TC11-18H10.1	Mouse	BV421	1:100	506925	Biolegend
IL-17f	eBio18F10	Mouse	Alexa Fluor 488	1:100	53.7471.82	eBioscience
IL-17f	9D3.1C8	Mouse	Alexa Fluor 488	1:100	517006	Biolegend
IL-22	IL22JOP	Mouse	APC	1:100	17-7222-82	Thermo Fisher
IFNγ	XMG1.2	Mouse	BV711	1:100	505835	Biolegend
IFNγ	XMG1.2	Mouse	APC	1:100	505810	Biolegend
IFNγ	XMG1.2	Mouse	PE/Cy7	1:100	505826	Biolegend
FoxP3	FJK-16S	Mouse/Rat	APC	1:100	17-5773	eBioscience
T-bet	4B10	Mouse/Human	PE/Cy7	1:100	644824	Biolegend
RORγt	Q31 378	Mouse	BV421	1:100	562894	BD
GATA3	16E10A23	Mouse/Human	Alexa Fluor 488	1:100	653808	Biolegend
pSTAT3 Y705	4/P-STAT3	Mouse/Human	Alexa Fluor 647	1:100	557815	BD

Table 2: Antibodies used for flow cytometry experiments

Probe	Cat no.	Exon Boundary
CD3ε	Mm00599684_g1	6-7
Tbp	Mm00446973_m1	4-5
Dhh	Mm01310203_m1	1-2
Shh	Mm00436528_m1	2-3
Ihh	Mm01259021_m1	1-2
Smo	Mm01162705_m1	2-3
Ptch1	Mm00436026_m1	17-18
Ptch2	Mm00436047_m1	19-20
Gli1	Mm00494654_m1	11-12
Gli2	Mm01293111_m1	13-14
Gli3	Mm00492345_m1	14-15
Il17a	Mm00439618_m1	1-2
Runx1	Mm01213404_m1	6-7
Rora	Mm01173766_m1	9-10
Rorct	Mm01261022_m1	5-6
Batf	Mm00479410_m1	1-2
Irf4	Mm00516431_m1	5-6
Vax2	Mm00496315_m1	1-2
Cd5l	Mm00437567_m1	5-6

Table 3: Probes used for qRT-PCR

Target	Clone	Reactivity	Type	Dilution	Cat no.	Supplier
Actin	AC-40	Mouse anti-human/rat/mouse	Primary	1:1’000	A3853	Sigma
Actin	AC-15	Mouse anti-human/rat/mouse	Primary	1:20’000	A5441	Sigma
AMPKα	N/A	Rabbit anti-human/rat/mouse	Primary	1:1’000	2532	Cell Signaling
pAMPKα Thr172	40H9	Rabbit anti-human/rat/mouse	Primary	1:1’000	2535	Cell Signaling
CaMKK2	D8D4D	Rabbit anti-human/rat/mouse	Primary	1:1’000	16810	Cell Signaling
LKB1	D60C5	Rabbit anti-human/rat/mouse	Primary	1:1’000	3047	Cell Signaling
Ptch	Polyclonal	Rabbit anti-human/mouse	Primary	1:1’000	ab53715	Abcam
Smoothened	18-10-10	rat	Primary	1:5 – 1:100 hybridoma supernatant	N/A	N/A
α-tubulin	DM1A	Mouse anti-human/rat/mouse	Primary	1:2’000	3873	Cell Signaling
HA	C29F4	Rabbit anti-HA	Primary	1:1000	3724	Cell Signaling
	Polyclonal	Goat anti-mouse	Secondary (HRP conj.)	1:15’000	P0447	Dako
	Polyclonal	Donkey anti-rabbit	Secondary (HRP conj.)	1:10’000	P0448	Dako
	Polyclonal	Goat anti-rat	Secondary (HRP conj.)	1:3’000	7077S	Cell Signaling
	Polyclonal	Goat anti-mouse	Secondary (IRDYE 680RD conj.)	1:15’000	926-68071	LiCor
	Polyclonal	Goat anti-mouse	Secondary (IRDYE 800CW conj.)	1:5’000	926-32210	LiCor

Table 4: Antibodies used for Western Blot
