# Supplementary material for: Cell-autonomous Hedgehog signaling controls Th17 polarization and pathogenicity
Source: Nat Commun. 2022 Jul 14;13:4075. doi: 10.1038/s41467-022-31722-5 (PMC9281293; doi:10.1038/s41467-022-31722-5)
Supplement: Supplementary file 3 — Reporting Summary [file 41467_2022_31722_MOESM3_ESM.pdf]

nature portfolio

Corresponding author(s): Maike de la Roche

Last updated by author(s): 10 June 2022

Reporting Summary

Nature Portfolio wishes to improve the reproducibility of the work that we publish. This form provides structure for consistency and transparency in reporting. For further information on Nature Portfolio policies, see our Editorial Policies and the Editorial Policy Checklist.

Statistics

For all statistical analyses, confirm that the following items are present in the figure legend, table legend, main text, or Methods section.

n/a	Confirmed	
☐	☒	The exact sample size (*n*) for each experimental group/condition, given as a discrete number and unit of measurement
☐	☒	A statement on whether measurements were taken from distinct samples or whether the same sample was measured repeatedly
☐	☒	The statistical test(s) used AND whether they are one- or two-sided<br>*Only common tests should be described solely by name; describe more complex techniques in the Methods section.*
☐	☒	A description of all covariates tested
☒	☐	A description of any assumptions or corrections, such as tests of normality and adjustment for multiple comparisons
☐	☒	A full description of the statistical parameters including central tendency (e.g. means) or other basic estimates (e.g. regression coefficient) AND variation (e.g. standard deviation) or associated estimates of uncertainty (e.g. confidence intervals)
☐	☒	For null hypothesis testing, the test statistic (e.g. *F*, *t*, *r*) with confidence intervals, effect sizes, degrees of freedom and *P* value noted<br>*Give P values as exact values whenever suitable.*
☒	☐	For Bayesian analysis, information on the choice of priors and Markov chain Monte Carlo settings
☒	☐	For hierarchical and complex designs, identification of the appropriate level for tests and full reporting of outcomes
☒	☐	Estimates of effect sizes (e.g. Cohen's *d*, Pearson's *r*), indicating how they were calculated

Our web collection on statistics for biologists contains articles on many of the points above.

Software and code

Policy information about availability of computer code

Data collection	GraphPad Prism 8 was used to input data and generate graphs. BD LSRII, BD LSR Fortessa, BD FACSymphony were used to aquire flow cytometry data. Confocal spinning disc microscopy was performed on an Andor Dragonfly 500 (Oxford Instruments) to acquire immunofluorescence data. RT-qPCR was performed on a QuantStudio 6 Flex Real-Time PCR System (Thermo Fisher). RNA-Seq was performed with the HiSeq4000 platform (Illumina). Western blots were developed by radiographic film or LiCor Odyssey CLx.
Data analysis	Data analysis was performed using GraphPad Prism 8. FlowJo Version 10.6.0 was used to analyse flow cytometry data. For RNA-Seq experiments, reads were aligned to the mouse genome version GRCm38 using STAR v2.5.3a. Read counts were obtained using feature Counts function in Subread v1.5.267 and read counts were normalized and tested for differential gene expression using the DESeq2 workflow Histological samples were analysed with AperioImageScope (Leica Biosystems). Immunofluorescence images were processed using Imaris software version 9.2, 9.6 and 9.7 (Bitplane/Oxford Instruments).

For manuscripts utilizing custom algorithms or software that are central to the research but not yet described in published literature, software must be made available to editors and reviewers. We strongly encourage code deposition in a community repository (e.g. GitHub). See the Nature Portfolio guidelines for submitting code & software for further information.

Data

Policy information about availability of data

All manuscripts must include a data availability statement. This statement should provide the following information, where applicable:

- Accession codes, unique identifiers, or web links for publicly available datasets
- A description of any restrictions on data availability
- For clinical datasets or third party data, please ensure that the statement adheres to our policy

The RNA-Seq data generated in this study have been deposited in NCBI's Gene Expression Omnibus under GEO Series accession number GSE205848 (https://www.ncbi.nlm.nih.gov/geo/query/acc.cgi?acc=GSE205848). Reads were aligned to the mouse genome version GRCm38 using STAR v2.5.3a. Publicly available data were obtained from GEO with the following accession numbers: GSE49877 (https://www.ncbi.nlm.nih.gov/geo/query/acc.cgi?acc=GSE49877), GSE109142 (https://www.ncbi.nlm.nih.gov/geo/query/acc.cgi?acc=GSE109142) and GSE117993 (https://www.ncbi.nlm.nih.gov/geo/query/acc.cgi?acc=GSE117993). Source data are provided with this paper.

Field-specific reporting

Please select the one below that is the best fit for your research. If you are not sure, read the appropriate sections before making your selection.

☒ Life sciences ☐ Behavioural & social sciences ☐ Ecological, evolutionary & environmental sciences

For a reference copy of the document with all sections, see nature.com/documents/nr-reporting-summary-flat.pdf

Life sciences study design

All studies must disclose on these points even when the disclosure is negative.

Sample size	Sample size was chosen according to typical standards in the field (PMID: 20493732, PMID: 30773462, PMID: 24101376) and the availability of novel GM mice.
Data exclusions	No animals were excluded from analysis in any experiment. For the analysis of the adoptive transfer colitis model only, data points from all three independent experiments were pooled and the presence of a single statistically significant outlier was assessed using Grubbs' test (alpha =0.05) and if present that single data point was excluded from further analysis.
Replication	Every experiment has been independently repeated at least 3 times within the main manuscript as detailed in the figure legends. Within the supplemental information 3 independent repeats were not always performed due to breeding problems in the GM lines (Suppl. Fig. 4b, 6,7d right panel).
Randomization	Genetic mouse experiments: Different genotypes of the same sex and from the same litter were compared where possible. Wild-type and Rag2KO recipient mice were randomly divided into control or treatment groups. For experiments performed on wildtype-derived cells (e.g. expression analysis, drug treatment etc.), wild-type cells were isolated, pooled and equally divided between the different experimental conditions.
Blinding	Pathology scores in the adoptive transfer colitis model were determined by a gastroenterologist who was blinded to experimental groups. Mice treatments were blinded to investigators at the time of harvest other than the lead investigator. For other experiments, blinding was not possible since the primary investigators performed the experiments from beginning to end due to the technical nature of the experiments.

Reporting for specific materials, systems and methods

We require information from authors about some types of materials, experimental systems and methods used in many studies. Here, indicate whether each material, system or method listed is relevant to your study. If you are not sure if a list item applies to your research, read the appropriate section before selecting a response.

Materials & experimental systems

n/a	Involved in the study	
☐	☒	Antibodies
☐	☒	Eukaryotic cell lines
☒	☐	Palaeontology and archaeology
☐	☒	Animals and other organisms
☒	☐	Human research participants
☒	☐	Clinical data
☒	☐	Dual use research of concern

Methods

n/a	Involved in the study	
☒	☐	ChIP-seq
☐	☒	Flow cytometry
☒	☐	MRI-based neuroimaging

Antibodies

Antibodies used | Antibodies for flow cytometry are detailed in Table 2 and for western blot detailed in Table 4.

Validation | For the novel Smo antibodies we have generated (18-10-10 and 18-2-3) validation is shown in Suppl. Fig. 3. Flow cytometry antibodies are well established clones and every lot is quality tested by the supplier using immunofluorescence staining. Each antibody was titrated for optimal performance and optimal concentrations are shown in the respective tables. All remaining antibodies were validated for the given species and application, either directly by the manufacturer or in the primary references cited on the manufacturer's website. These citations are visible on the manufacturer websites for each specific antibody (details of catalogue numbers see respective tables in the "methods" section).

Eukaryotic cell lines

Policy information about cell lines

Cell line source(s) | Smo KO mouse embryonic fibroblasts (MEFs) were generated by James Chen (Stanford University). WT MEFs were a generous gift from Jane Goodall (University of Cambridge). HEK 239T cells were a generous gift from James Brenton (University of Cambridge) – nota bene these cells were not used directly to generate any experimental data, they were used solely for the generation of retroviral supernatant.

Authentication | Cell lines are authenticated by in-house STR analysis.

Mycoplasma contamination | All cell lines tested mycoplasma negative (MycoProbe Mycoplasma Detection Kit, R&D systems).

Commonly misidentified lines (See ICLAC register) | No commonly misidentified cell lines were used in this study.

Animals and other organisms

Policy information about studies involving animals; ARRIVE guidelines recommended for reporting animal research

Laboratory animals | All mice were on the C57BL/6J background. We used both male and female mice for all experiments between 6 weeks and 6 months of age. Mice were housed under specific-pathogen free conditions at the University of Cambridge, Cancer Research UK Cambridge Institute. Mouse housing conditions were in a 12 h light-dark cycle at room temperature (20–23 °C) with 40–70% humidity with ad libitum access to food and filtered water. Environmental enrichment, such as mouse houses or fun tunnels and nesting material were provided to all animals. Details of the specific GM lines can be found in the methods section.

Wild animals | No wild animals were used in this study.

Field-collected samples | No field collected samples were used in this study.

Ethics oversight | All housing and procedures were performed in strict accordance with the United Kingdom Home Office Regulations, the Danish Council for Animal Testing/The Supervisory Authority on Animal Testing, and the Cancer Research UK Cambridge Institute Animal Welfare and Ethical Body (AWERB).

Note that full information on the approval of the study protocol must also be provided in the manuscript.

Flow Cytometry

Plots

Confirm that:

- [x] The axis labels state the marker and fluorochrome used (e.g. CD4-FITC).
- [x] The axis scales are clearly visible. Include numbers along axes only for bottom left plot of group (a 'group' is an analysis of identical markers).
- [x] All plots are contour plots with outliers or pseudocolor plots.
- [x] A numerical value for number of cells or percentage (with statistics) is provided.

Methodology

Sample preparation | Murine naive CD4 T cells were purified from the spleen as detailed in the materials and methods. CD4 T cell populations from in vivo experiments were purified from the spleen, intestine and mesenteric Lymph node as detailed in the materials and methods.
Surface staining
Cells were stained in 96-well round-bottom plates (Corning) or 5 ml polystyrene round bottom tubes (Fisher Scientific/Falcon, cat no. 352003). Samples were washed twice with ice-cold PBS and incubated for 10 min light protected at room temperature with Fixable Viability dye eFlour780, prepared at 1:1000 in PBS (eBioscience, cat no. 65-0865-18; 500 tests). Cells were then washed once with FACS buffer, made with PBS (Gibco, cat no. 14190094) + 3% FCS (Biosera) + 0.05% Sodium Azide (Sigma, cat no. 71289), + 2 mM EDTA (prepared in-house). Cells were then incubated with Fc block (1:100; Biolegend TruStain fcX anti-mouse CD16/32, cat no. 101320) for 5 min at room temperature protected from light. Next, cells were

incubated with 50 µl of fluorophore-conjugated antibodies at the appropriate dilution (Table 1) for 20 min at 4oC protected from light. Cells were washed twice with FACS buffer and moved to 5 ml polystyrene round bottom tubes prior to immediate analysis (Fisher Scientific/Falcon) or fixation for intracellular staining.

Intracellular staining

For the staining of intracellular cytokines, cells were incubated at 37°C for 4h in the presence of 1 µg/ml Ionomycin (Sigma, cat no. I9657) and 50 ng/ml PMA (Sigma, cat no. P1585) and Golgistop (BD, Cat no. 554724) added for the duration of stimulation per the manufacturer's instructions. Following surface marker staining (as above), cells were fixed with BD Cytofix/Cytoperm Plus Fixation Buffer (BD Biosciences, cat no. 554715) for 25 min at 4°C protected from light. Samples were then spun down in a table-top centrifuge and washed once with permeabilization buffer (10x; BD Biosciences, cat no. 554715), prepared to a final concentration of 1x with Milli-Q Water (in-house). Cells were resuspended in 50 µl of permeabilization buffer containing fluorophore-conjugated antibodies at the appropriate concentration (Table 1) and incubated light protected at 4°C for 30 min. Prior to analysis, cells were washed once in permeabilization buffer and once in FACS buffer.

Instrument

BD LSRII or BD LSR Symphony cell analyzers or a BDAria sorter was used.

Software

FlowJo software (Tree Star Inc., version 10.4) was used for all flow cytometry data analysis.

Cell population abundance

Information about cell population abundance is in the paper and in the methods.

Gating strategy

Gating strategies were utilized according to standard practice for flow cytometry. Gating strategies for the in vivo experiments are shown in supplemental figures.

☒ Tick this box to confirm that a figure exemplifying the gating strategy is provided in the Supplementary Information.
